# Tuberculosis associated with the living conditions in an endemic municipality in the North of Brazil*

**DOI:** 10.1590/1518-8345.3223.3343

**Published:** 2020-08-31

**Authors:** Suzana Rosa André, Laura Maria Vidal Nogueira, Ivaneide Leal Ataíde Rodrigues, Tarcísio Neves da Cunha, Pedro Fredemir Palha, Claudia Benedita dos Santos

**Affiliations:** 1 Universidade do Estado do Pará, Escola da Enfermagem Magalhães Barata, Departamento de Enfermagem Comunitária, Belém, PA, Brazil.; 2 Scholarship holder at the Coordenação de Aperfeiçoamento de Pessoal de Nível Superior (CAPES), Brazil.; 3 MICROARS Consultoria e Projetos, Programa Nacional de Cooperação Acadêmica da Coordenação de Aperfeiçoamento de Pessoal de Nível Superior (CAPES), Rio de Janeiro, Rio de Janeiro, Brazil.; 4 Universidade de São Paulo, Escola de Enfermagem de Ribeirão Preto, PAHO/WHO Collaborating Centre at Nursing Research Development, Departamento de Enfermagem Materno-infantil e Saúde Pública, Ribeirão Preto, SP, Brazil.

**Keywords:** Tuberculosis, Spatial Analysis, Geographic Information Systems, Epidemiology, Incidence, Quality of Life, Tuberculose, Análise Espacial, Sistemas de Informação Geográfica, Epidemiologia, Incidência, Qualidade de Vida, Tuberculosis, Análisis Espacial, Sistemas de Información Geográfica, Epidemiología, Incidencia, Calidad de Vida

## Abstract

**Objective:**

to analyze the association between the occurrence of new tuberculosis cases and the Adapted Living Condition Index, and to describe the spatial distribution in an endemic municipality.

**Method:**

this is an analytical and ecological study that was developed from new cases in residents of an endemic municipality in the North Region of Brazil. The data were obtained from the Notifiable Diseases Information System and from the 2010 Demographic Census. The Adapted Living Conditions Index was obtained by factor analysis and its association with the occurrence of the disease was analyzed by means of the chi-square test. The type I error was set at 0.05. Kernel estimation was used to describe the density of tuberculosis in each census sector.

**Results:**

the incidence coefficient was 97.5/100,000 inhabitants. The data showed a statistically significant association between the number of cases and socioeconomic class, with the fact that belonging to the highest economic class reduces the chance of the disease occurring. The thematic maps showed that tuberculosis was distributed in a heterogeneous way with a concentration in the Southern region of the municipality.

**Conclusion:**

tuberculosis, associated with precarious living conditions, reinforces the importance of discussion on social determinants in the health-disease process to subsidize equitable health actions in risk areas, upon a context of vulnerability.

## Abstract (pt)

**Objetivo:**

analisar a associação entre a ocorrência dos casos novos de tuberculose e o Índice Adaptado de Condição de Vida e descrever a distribuição espacial em um município endêmico.

**Método:**

estudo analítico, ecológico desenvolvido a partir dos casos novos em residentes de um município endêmico da região Norte do Brasil. Os dados foram obtidos do Sistema de Informação de Agravos de Notificação e do Censo Demográfico 2010. O Índice Adaptado de Condição de Vida foi obtido por análise fatorial e sua associação com a ocorrência da doença foi analisada pelo teste qui-quadrado. Fixou-se erro tipo I igual a 0,05. Foi utilizada estimativa Kernel para descrever a densidade da tuberculose por setor censitário.

**Resultados:**

o coeficiente de incidência foi 97,5/100.000 habitantes. Os dados mostraram associação estatisticamente significativa entre número de casos e classe socioeconômica, sendo que pertencer à classe econômica mais alta reduz a chance de ocorrência da doença. Os mapas temáticos mostraram que a tuberculose se distribuiu de forma heterogênea com concentração na região sul do município.

**Conclusão:**

a tuberculose, associada às condições de vida precárias, reforça a importância de discussão sobre determinantes sociais no processo saúde-doença, para subsidiar ações equânimes de saúde nas áreas risco, mediante contexto de vulnerabilidade.

## Abstract (es)

**Objetivo:**

analizar la asociación entre la incidencia de nuevos casos de tuberculosis y el Índice Adaptado de Condición de Vida y describir la distribución espacial en un municipio endémico.

**Método:**

estudio analítico y ecológico desarrollado a partir de nuevos casos en residentes de un municipio endémico en el Norte de Brasil. Los datos se obtuvieron del Sistema de Información de Enfermedades de Notificación Obligatoria y del Censo Demográfico 2010. El Índice Adaptado de Condición de Vida se obtuvo mediante análisis factorial y su asociación con la incidencia de la enfermedad se analizó mediante la prueba de chi-cuadrado. Se fijó el error tipo I igual a 0,05. La estimación de Kernel se utilizó para describir la densidad de tuberculosis por sección censal.

**Resultados:**

el coeficiente de incidencia fue de 97,5/100.000 habitantes. Los datos mostraron una asociación estadísticamente significativa entre el número de casos y la clase socioeconómica, siendo que pertenecer a la clase económica más alta reduce la posibilidad de desarrollar la enfermedad. Los mapas temáticos mostraron que la tuberculosis se distribuía de manera heterogénea, con mayor concentración en la región sur del municipio.

**Conclusión:**

la tuberculosis, asociada con condiciones de vida precarias, refuerza la importancia de discutir los determinantes sociales en el proceso de salud-enfermedad, a fin de subsidiar acciones de salud equitativas en áreas de riesgo, a través del contexto de vulnerabilidad.

## Introduction

Tuberculosis (TB) is an infectious disease which is caused by *Mycobacterium tuberculosis*, with a high impact on global public health^(^ 1 ^)^. It is a millennial, single-agent disease that has caused the most fatalities and affects thousands of people around the world, ranking among the 10 diseases with the highest mortality rates on the planet^(^ 2 ^-^ 3 ^)^.

For 2018, 10 million new cases were estimated in the world, with an incidence in the countries varying from 5 to more than 500 cases *per* 100,000 inhabitants. The highest concentration of cases occurred in Southeast Asia (44%), Africa (24%), and the Western Pacific (18%), and with smaller proportions in the Mediterranean region (8%), the Americas (3%), and European countries (3%)^(^ 3 ^)^.

Brazil is among the 30 countries with the highest TB loads^(^ 3 ^)^. For 2019, the incidence coefficient was 35.0 cases *per* 100,000 inhabitants. The incidence in the country had decreased between 2010 and 2016 but, from 2017 to 2018, this measure increased^(^ 4 ^)^. In 2019, the state of Pará was among the federal units with an incidence rate close to or above the national coefficient, and its capital city, Belém, was among the 5 capitals with most incidence of the disease in 2018 (62.7 cases/100,000 inhab.)^(^ 4 ^-^ 5 ^)^.

The epidemic in the country does not have a heterogeneous character, but it has centralized on vulnerable populations such as street people, individuals deprived of their freedom, indigenous population, and individuals living with the Human Immunodeficiency Virus (HIV). In this sense, clinical and epidemiological management is a challenge for health professionals, managers, TB patients, families, and organized civil society to implement inclusive, focused, and co-accountability policies^(^ 3 ^-^ 6 ^)^.

There are several factors that boost the occurrence of TB, among them, the socioeconomic conditions and the difficulties of access to the health services. Such conditions express the precarious living conditions related to poverty, low schooling, unhealthy housing, population thickening, and abusive drug use^(^ 7 ^)^.

In this sense, TB has been considered a marker of social inequities in health^(^ 8 ^)^. The persistence of unequal social models interferes in the health-disease process, especially in the chain of transmissibility, and it predicts the multi-causal dynamics of illness based on the social determinants of health, regarding low living conditions and its impact on the individual/society relationship in the different regions of the country.

In order to provide satisfactory answers on the density of TB and its distribution in relation to the living conditions, this study proposes a smoothed innovative spatial analysis, independent of the geographical limits for the visualization of the disease. Studies that consider the spatial and temporal diffusion of diseases make it possible to understand how the occurrence of health adverse events affects population groups and spread in territories^(^ 9 ^)^.

It is understood that a broad look at the needs of the population can support public policies and guidelines for planning actions and conducting Primary Health Care services, based on emancipatory practices, aimed at attaining global goals to combat TB.

Thus, the hypothesis of this study is that the occurrence of new TB cases is associated with the strata of the municipality with more precarious living conditions, and the objectives are the following: to analyze the association between the occurrence of new tuberculosis cases and the Adapted Living Condition Index, and to describe the spatial distribution in an endemic municipality.

## Method

An ecological and analytical study conducted in the city of Belém, PA. This research is part of the project entitled “Space-Time association among neglected diseases and the Living Condition Index: Identification of priority areas for the implementation of active methodologies in public schools as a health education strategies” of the National Program for Academic Cooperation (*Programa Nacional de Cooperação Acadêmica*, PROCAD)/CAPES Edict No. 071/2013. Belém is located in the North Region of Brazil, in the Amazon biome, with a total population estimated of 1,452,275 inhabitants in 2017, with a total territorial area of 1,059,458 km^2(^ 10 ^)^.

The population of this study consisted of the new TB cases (incidence) notified to the Notifiable Disease Information System (*Sistema de Informações de Agravos de Notificações*, SINAN) in the period from 2009 to 2016, in individuals residing in the urban and rural areas of the municipality of Belém at the time of the diagnosis. This region was chosen as it is a priority for TB control due to its high incidence and to the existence of precarious housing settlements, and because it has a significant representation of the indigenous population, which is considered as vulnerable by the public policies.

To characterize the participants, the following variables were selected: age, gender, schooling, clinical TB form and HIV serology. The spatial distribution of TB was based on the addresses of the new cases obtained from a single SINAN spreadsheet.

The variables that made up the construction of the Adapted Living Condition Index (ALCI) originated from the 2010 Demographic Census of the Brazilian Institute of Geography and Statistics (*Instituto Brasileiro de Geografia e Estatística*, IBGE), using the methodology proposed by a research study conducted in the city of Recife on living conditions associated with infant mortality^(^ 11 ^)^. The unit of analysis for building the Index was the census sector. This study considered the following variables for the construction of the ALCI: the proportion of homes without adequate water supply, homes without adequate sanitary installations, the proportion of homes without direct garbage collection, the proportion of the 10-14-year-old illiterate population, the proportion of home heads with four years or less of study, heads of homes with a monthly income less than or equal to two minimum wages, and in-bedroom density.

The information was organized in spreadsheets in order to build a Geographic Database (*Banco de Dados Geográfico*, BDGeo) debugged by means of the *Microsoft Office Excel* ^®^2010 software. The data analysis was carried out in two stages, in the first one statistical data were described with measures of position (mean, mode, median), dispersion (variance, standard deviation), and variability added to the epidemiological indicators. In the second, the inferential process was carried out using the technique of spatial analysis of the cases that allowed for the visualization of risk areas for TB and for conducting the tests of association between the number of new TB cases and the levels of living condition, elaborated from cutoff points in the values of the ALCI, according to cluster analysis.

To build the ALCI, the factorial analysis, according to the technique of the main components, allowed stratifying the municipality by means of scores produced by the Statistical Program for the Social Sciences (SPSS), version 23.0. This technique produces factor regression coefficients (loadings) indicating the relationship between the factor and each original variable, determining the percentage of the total variance explained for each extracted factor^(^ 11 ^)^.

For the production of living condition strata, the ALCI used the hierarchical cluster analysis grouping technique, identifying 4 strata. This cluster analysis is a multivariate classification technique that aims to group data according to their similarities^(^ 12 ^)^. To verify the association between the four levels of living condition elaborated from cutoff points in the ALCI values, according to cluster analysis and the number of new TB cases, the Chi-square test was performed. For comparison between the occurrence of TB cases and the socioeconomic stratum, estimates of the Odds Ratio values and respective confidence intervals were obtained. To obtain the estimates, the R Core Team 2018 program^(^ 13 ^-^ 14 ^)^ was used. The type I error was set at 0.05 (α = 0.05).

The association analyses were chosen due to the categorical nature of the living condition level variable (Stratum I: Low living condition; Stratum II: Medium - low living condition; Stratum III: Medium - high living condition; Stratum IV: High living condition) and to the dichotomous nature of the occurrence of TB cases variable (counting data).

For mapping, analysis of the data’s spatial behavior, and geocoding of the addresses, vector files were initially obtained from the digital cartographic bases, by meshes of the census sector of Belém. Subsequently, the formatting and spelling correction of the spreadsheet with the addresses of the TB cases notified *per* residence were performed. It should be noted that, at this stage, under-notifications, errors in data entries, and failure to handle the Information System may have caused geocoding losses.

Finally, the addresses were geocoded in the Universal Transverse Mercator (UTM) projection, zone 20, where Belém is located, through a batch-type geocoding website that uses the Google Earth^®^ database, called “doogal.co.uk” (https://www.doogal.co.uk/BatchGeocoding.php). The geographical analyses were performed with the TerraView 4.2.2 app from the National Institute of Space Research (*Instituto Nacional de Pesquisas Espaciais*, INPE)^(^ 15 ^)^.

To estimate a territorial distribution surface of TB from geocoded addresses, the Kernel Density Estimator (KDE) was used. The main objective of the KDE is to generate a regular grid where each cell represents a density value^(^ 16 ^)^. This is a non-parametric technique that promotes statistical smoothing, giving rise to chromatic gradients with “hot areas” to the extent that in that region there is a vast density of cases^(^ 16 ^)^. The KDE method is based on search radios that can be prefixed or adaptive. Due to the unequal distribution of the cases, the quartic-function adaptive radio was used.

Cluster detection techniques tend to have a spatial distribution similar to the population distribution in the health events. This distribution may derive from social, historical, and economic organizations. However, the Kernel estimator does not predict only the distribution of clusters but explores the behavior pattern of the health data points. Thus, it generates a continuous surface from point data, which allows for a quick visualization of the areas that deserve more attention, being an important tool for the analysis of events and for the rapid implementation of strategies in the area of public health^(^ 17 ^)^.

This study was approved by the Research Ethics Committee of the Undergraduate Nursing Course of the University of the State of Pará, under opinion No. 2,279,847.

## Results

Between 2009 and 2016, 11,103 new TB cases were notified in Belém with an incidence coefficient of 97.5/100,000 inhabitants, mean age of 38.6 years old, and a standard deviation of 17.1. Throughout this period, the incidence coefficient of TB was higher in men (12.4/10,000 men) than in women (7.3/10,000 women), and the age group most affected was that of the older adults aged 60 or over (13.8/10,000).

To identify possible social disparities in the geographic space of Belém, the ALCI was built with seven variables, whose data were obtained in the IBGE electronic portal, using descriptive statistics (Table 1).

**Table 1:** Descriptive statistics of the variables used to obtain the ALCI*. Belém, PA, Brazil, 2018

Variable (%)	Mean	Standard deviation	Median	Maximum value	Minimum value	Losses
In-bedroom density	3.79	0.38	3.81	5.79	2.37	1
Heads of home with a monthly income less than or equal to two minimum wages	69.66	23.41	78.67	101.69	2.50	1
Homes without adequate sanitary installations	61.16	32.08	64.47	100	0	1
The proportion of homes without direct garbage collection	3.54	12.54	0	100	0	0
The proportion of homes without adequate water supply	23.79	28.42	11.68	100	0	1
The proportion of the 10-14-year-old illiterate population	2.93	3.18	2.20	30.77	0	0
The proportion of heads of home with four years or less of study	4.13	3.95	3.15	30.77	0	0

Source: Brazilian Institute of Geography and Statistics (*Instituto Brasileiro de Geografia e Estatística*, IBGE)

* ALCI = Adapted Living Condition Index

It was identified that the in-home density had a mean of 3.79% individuals *per* bedroom, with a maximum of 5.79% and a minimum of 2.37%. For the “heads of home with a monthly income less than or equal to two minimum wages” variable, the mean was 69.66%, with a maximum value of 101.69% and a minimum of 2.5%.

For the “the proportion of homes without sanitary exhaustion”, “the proportion of homes without garbage collection”, and “the proportion of homes without adequate water supply” variables, the means were 61.1%, 5.53%, and 23.8%, respectively. The mean for the illiterate population aged 10-14 years old was 2.93%, showing a significant and diversified variation of the population, ratified by the mean for heads of home with four years or less of study (4.15%).

The variables that composed the ALCI showed statistically significant positive or negative linear correlations among them. Based on these results, the option was to use factor analysis considering only one dimension, which allowed identifying the contribution of the secondary variables and the main factor of the living conditions in Belém (Table 2).

**Table 2:** Matrix of the index loads related to the living conditions. Belém, PA, Brazil, 2018

Variables	Factorial Loads (loadings)
The proportion of heads of home with four years or less of study	0.88
Heads of home with a monthly income less than or equal to two minimum wages	0.79
The proportion of the 10-14-year-old illiterate population	0.72
Homes without adequate sanitary installations	0.51
The proportion of homes without direct garbage collection	0.48
In-bedroom density	0.42
The proportion of homes without adequate water supply	0.26

Source: Brazilian Institute of Geography and Statistics (*Instituto Brasileiro de Geografia e Estatística*, IBGE)

The variables that expressed higher factor loads were the following: the proportion of heads of home with four years or less of study (0.88); heads of home with a monthly income less than or equal to two minimum wages (0.79); and the proportion of the 10-14-year-old illiterate population (0.72).

Based on the results obtained, the stratification of the municipality was carried out (hierarchical cluster analysis), dividing the findings into 4 clusters, namely: low living condition (I), medium-low living condition (II), medium-high living condition (III), and high living condition (IV).

The chi-square test showed a statistically significant association between the occurrence of TB cases and socioeconomic class (χ^2^_3; 0.05_= 104.51; p < 0.001).

Table 3 presents the estimates of the Odds Ratios (ORs), respective Standard Deviations (SDs), values for the Normal Standardized (z) variable, statistical significance p(>|z|), and intervals with 95% confidence (IC [95%]) obtained for the cases of Tuberculosis outcome, by clusters according to the ALCI.

**Table 3:** Estimates of the Odds Ratios, respective Standard Deviations, values for the Normal Standardized variable, statistical significance, and 95% confidence intervals obtained for the cases of tuberculosis outcome, by clusters according to the Adapted Living Condition Index. Belém, PA, 2018

Clusters according to ALCI*	TB cases^†^	Rate/ Thousand inhab.	OR^‡^	SD^§^	Z^||^	p (Z>׀z׀)^¶^	[95%]CI**	
I	1,657	5.1	1.22	0.12	10.56	< 0.00001	1.01	1.47
II	3,442	6.5	1.56	0.14	10.74	< 0.00001	1.30	1.87
III	2,714	5.3	1.28	0.12	10.70	< 0.00001	1.06	1.53
IV	120	4.2	Ref^††^	-	-	-	-	-

* ALCI = Adapted Living Condition Index;

† TB = Tuberculosis;

‡ OR = Odds Ratios;

§ SD = Respective Standard Deviations;

|| Z = Values for the Normal Standardized variable;

¶ p (Z>׀z׀) = Statistical significance;

** [95%]CI = 95% confidence intervals obtained for the outcome;

†† Ref = Reference Class

The values for the Odds Ratios (ORs) and their respective confidence intervals show that the chances of TB cases in the III, II and I strata are increased by approximately 28%, 56%, and 22%, respectively when compared to stratum IV.

**Figure 1:**
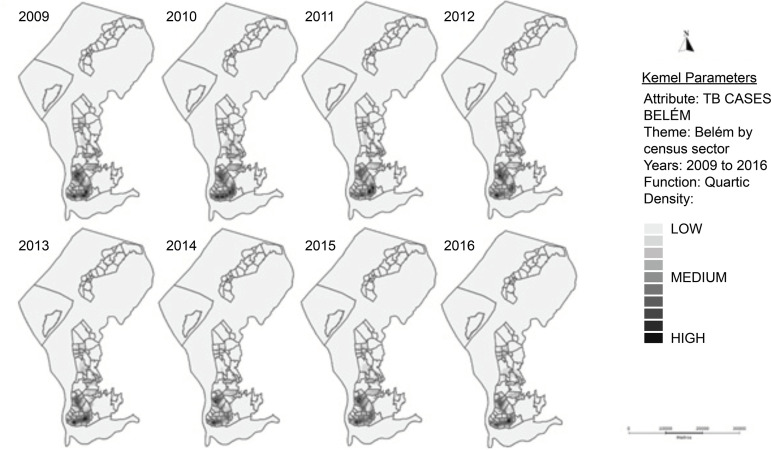
Maps with density distribution of new tuberculosis cases, of residents in the municipality, obtained through the Kernel Density Estimator for the studied period. Belém, PA, 2018

For spatial analysis, 7,957 (71.7%) cases were geocoded, since 3,146 (28.3%) presented failures in the geocoding due to inconsistency in the addressing system. From the geocoding it was possible to produce Kernel maps, expressing the density of TB cases, which is higher in the darker regions (Figure 1).

During the study period, TB presented a profile with similar incidence areas over the years with regard to the geographic regions affected; nevertheless, the density varied at the level of the census sectors and consequently in the visualization of the density pattern in the neighborhoods. The thematic maps expressed the high density of the disease in the neighborhoods of Terra Firme, Guamá, Cremação, Jurunas, Pedreira, Telégrafo, Sacramenta, and Barreiro in all the years studied.

It should be noted that in the neighborhood of Canudos, from 2009 to 2011, there was a high density of TB cases, mainly in regions bordering with other neighborhoods; however, in the following years, from 2012 to 2016, there was a decrease in the density of cases both in Canudos and in the neighborhoods closer to it. In 2016, only the Guamá neighborhood showed a higher density compared to the other neighborhoods in the city of Belém.

**Figure 2:**
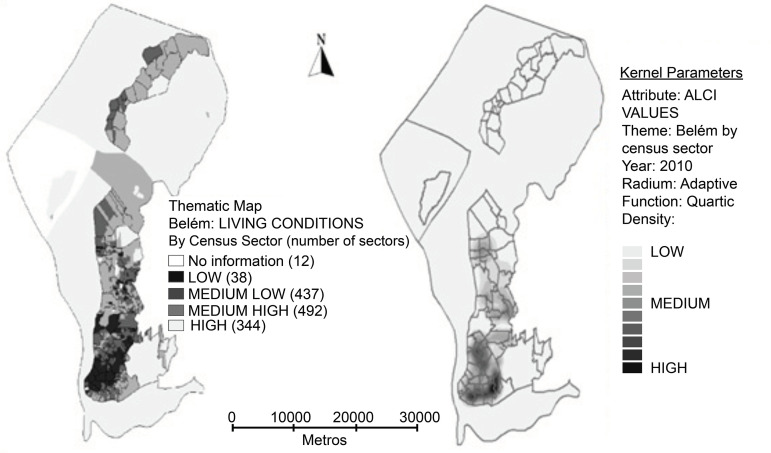
Thematic map of the 4-level stratified Adapted Living Condition Index (ALCI) compared to the Kernel density. The neighborhood boundary layer was overlaid as a territorial reference. Belém, PA, 2010

To visualize this association, the digital map of census sectors stratified according to the ALCI was obtained. The thematic map in Figure 2 shows the stratification of Belém according to the living conditions, analyzed through census sectors with overlapping neighborhoods, where the regions with darker shades represent worse or lower living conditions, while the lighter shades represent better living conditions.

From Figure 2 it was possible to identify a heterogeneous case density similar to the clusters obtained for the years 2009 to 2016 (Figure 1) through the KDE, and it was possible to visualize the relationship of TB with poorer and denser regions in population terms, but also reaching areas considered intermediate of the municipality.

## Discussion

The results of this study showed a spatial dependence in the occurrence of TB cases, with higher density in the Southern region of this municipality, over the years studied. It is important to consider that the tendency of TB is associated with multiple historical and social processes that involve social determinants of the health-disease process and demand individual, collective, and programmatic strategies of the social actors to eliminate it, especially in vulnerable populations^(^ 18 ^-^ 19 ^)^.

The results showed greater predominance of cases in young adults and in individuals over 60 years of age, with 13.3 cases/100,000 inhabitants in the 20-29 age group, and 13.8 for those over 60 years of age. This profile was similar in the research conducted in the states of Pará and Piauí, where the mean age of the TB patients was 35.3 years old, following the national tendency of the disease in the age group from 20 to 49 years old, compromising the most productive phases of the lives of the patients, which characterizes the disease as a social and economic problem^(^ 20 ^-^ 21 ^)^.

The pulmonary clinical form was the most frequent, as well as in other scenarios in Brazil^(^ 21 ^-^ 23 ^)^. Most of the cases resulted in a negative diagnosis for HIV/TB co-infection, corroborating another study conducted in the Northern region of the country^(^ 21 ^)^.

Over the period, the incidence coefficient of TB was higher in men (12.4/10,000 men) than in women (7.3/10,000 women). For this aspect, the relationship of TB with the male social network inserted in the studied territory must be considered. The crowded places where men most frequently go, for example bars, games, and parties, may favor transmissibility. In addition, access to the health services for these men is limited due to the incompatibility of their working hours with the operation of the services^(^ 24 ^)^. Thus, actions for an active search and health education strategies are needed in the men’s social network, as well as the viability of compatible schedules in the health services.

The results showed that patients who did not complete elementary school represented a significant portion of the cases in this study (32.9%). Individuals with low levels of education and in poor socioeconomic conditions have less chance of perceiving the risks of TB transmission, showing little commitment to the specific treatment, which is a consequence of restricted access to information, knowledge benefits, consumer goods, and health services^(^ 25 ^)^.

A higher prevalence of the pulmonary clinical form was observed, which characterizes a higher risk of transmissibility among the people living in the studied municipality, due to its high infectivity. The interruption of transmission requires immediate intervention by the health services to promptly diagnose and treat the disease^(^ 21 ^)^, in addition to notification and active search for the patients’ contacts. Each patient diagnosed with TB tends to infect 10 to 15 people within a year, and one or two get ill, maintaining the cycle of the endemic^(^ 26 ^)^.

Individuals living with HIV are 30 times more likely to develop TB than those not infected with the virus, so testing for HIV is considered the standard for people with TB; however, coverage is still restricted in the health services, which demonstrates the opportunistic and lethal potential of TB for patients living with HIV/AIDS^(^ 21 ^-^ 27 ^)^.

TB and HIV/AIDS underreporting can be a limiting factor in the estimation of co-infection, not accurately demonstrating the real dimension of the problem, inferring as one of the causes of the slowness in the release of the results and of outdated databases in the Municipal Health Secretariats^(^ 21 ^,^ 25 ^)^.

The results also show that the “heads of home with a monthly income less than or equal to two minimum wages” variable portrays the precarious socioeconomic panorama of the population in Belém, since it presented a mean of 69.6%, while in municipalities like Ribeirão Preto this mean was 23%, revealing the influence of the economic factor as a conditioning factor for the development of TB in low-income individuals^(^ 28 ^)^.

Another relevant aspect for the city of Belém was the relation of the “homes without adequate sanitary installations” variable, with a mean value of 61.1%, while the IBGE, in a classification given by the 2010 Demographic Census, concludes that sanitary exhaustion was adequate in 67.9% of the homes^(^ 10 ^)^.

TB is associated with low living conditions and income, related to problems such as population growth, street people, chemical dependency, poor housing conditions, poor nutrition, low income, lack of sanitation, and other determinants^(^ 29 ^)^.

The ALCI obtained the “picture” of the living conditions of the population since the variables studied concern the socioeconomic aspects related to TB, which, even associated with underprivileged conditions, reach expressively strata with better living conditions^(^ 9 ^)^.

The result observed for class I may be related to the underreporting of cases in this stratum of the population. Individuals with low levels of education and in poor socioeconomic conditions have less chance of perceiving the risks of TB transmission, showing little commitment to the specific treatment, which is a consequence of restricted access to information, knowledge benefits, consumer goods, and health services^(^ 27 ^)^.

The location and geographical analysis of areas considered at risk for TB development were presented in this study through spatial analysis techniques, which contributed to the understanding of the current health context and its trends, building approaches directed at health surveillance practices, such as the identification of risk areas, population concentration, and prioritization of actions and resources, as well as the possible association of local conditions in the social environment where the patients live^(^ 30 ^)^.

A study conducted in the municipality of Belém showed that the spatial analysis exhibited areas with similar TB incidence with a tendency to clusters, and the same profile was found in this study, where neighborhoods with similar TB rates were close by. Although the distribution density is given in a variety of ways, the geographic regions affected by TB showed a predictable distribution pattern regarding the affected neighborhoods, which leads to questions about the effectiveness of disease control actions in these places^(^ 31 ^)^.

A study conducted in Ethiopia concluded that, despite different intervention programs aimed at reducing disease transmission and improving diagnosis, abnormal incidence rates persisted in the same locations with the most likely spatial clusters^(^ 32 ^)^. In Belém, the TB spatial clusters demonstrated, year by year, a stable pattern of territorial features, suggesting as a possible explanation that the intervention process may not be adequately focused on social determinants directly associated with the epidemiology of the disease.

Another research conducted in Madagascar presented a profile similar to the findings of this study, where the spatial aggregation zones of TB in the urban municipality have not changed substantially since previous surveys carried out at the research site, associating the high demographic density of the urban municipality with the high occurrence of the disease^(^ 33 ^)^.

Regarding the clusters formed by the association between TB and the ALCI, it can be highlighted in this study that the municipality of Belém presented similar characteristics to the findings in the literature^(^ 28 ^,^ 34 ^)^.

The higher concentration of TB in strata of poorer living conditions shows that the disease is associated with underprivileged conditions; however, even in strata of better living conditions a significant number of cases of the disease are still found^(^ 9 ^)^. Findings of a research conducted in Campina Grande revealed a higher mean incidence rate in strata of “worse” living conditions; however, the “best” living condition stratum had a higher incidence rate than the “regular” and “bad” living condition strata^(^ 9 ^)^. Understanding the ways in which the disease spreads and how health actions are implemented impacts on the planning measures focused on the diversities.

The planning actions to combat and control TB must be assured so that the health service is prepared, offering quality and accessible assistance that presents better health outcomes throughout the country^(^ 35 ^)^. The interventions should be targeted at underprivileged areas as these are regions most affected by the disease, in order to reduce transmission^(^ 36 ^)^.

In a study conducted in South Africa, the distance to the diagnostic health unit in a cohort of patients with resistant TB was assessed, and it was found that a large proportion of patients sought the health service outside their home district^(^ 37 ^)^. Such a situation may reveal the stigma related to the disease still present in society, which is capable of contributing to the low adherence to the treatment and the search for services outside the area of their territory.

This perspective reinforces the need for a broad offer of health services in a universal and capillary manner in the social environment, in addition to health surveillance with effective prevention, diagnosis, and treatment strategies aimed at combating the disease^(^ 20 ^)^.

This study may contribute to a critical reflection on the living conditions and on the aspects that make up the social relations of strength that produce and reproduce ineffective models of combating this endemic. In view of this, it is suggested that other studies be carried out which consider the social dynamics of the municipality, given the reality of the lives of its residents.

A limitation of this study stems from the use of secondary data from the database made available by the Municipal Health Secretariat, in spite of all the improvement efforts that have been undertaken. In this official database, fed by typing, it is important to complete the notification form correctly. Many study limitations stem from insufficient data completion and reporting, resulting mainly in incompleteness, underreporting, incorrect records, and information losses^(^ 21 ^,^ 38 ^-^ 39 ^)^.

More importantly, the spatial analysis was based on the geocoding of the cases by addresses obtained from the aforementioned database, with inevitable losses due to absences and inconsistencies.

Since one of the study objectives was to correlate TB with ALCI in the urban space, no population-based rates were calculated; however, the spatialization of the ALCI is done by Census Sector. Hence the adoption of the density estimator by Kernel to infer the correspondence of TB occurrence with the ALCI. It is suggested that this study be extended in the future to bring analyses by a spatial structure such as Census Sector or Neighborhood.

Additionally, we signal the difficult separation into lower economic class strata in a municipality where the majority of the population is socially segregated, but with territorial concentrations not always clear. In order to highlight the territorial discrimination in these cases, more refined indicators would be needed, taking into account other aspects of the social inequalities.

## Conclusion

The description of the TB spatial pattern allowed the intensity of the disease to be visualized from the behavior of point patterns, which constitutes a refined first-order analysis to subsidize equitable public health actions in risk areas.

In addition, the statistically significant association between the occurrence of TB and the strata representing worse living conditions reasserts that the disease remains associated with social vulnerability reaching more people in situations of exclusion. These findings reinforce the importance to effectively discuss the health social determinants, which are essential for planning and formulating intervention measures for combating and controlling the disease in this context.
